# Supplementary material for: Functional Analysis of the PCCA and PCCB Gene Variants Predicted to Affect Splicing
Source: Int J Mol Sci. 2021 Apr 16;22(8):4154. doi: 10.3390/ijms22084154 (PMC8073151; doi:10.3390/ijms22084154)
Supplement: Supplementary file 1 [file ijms-22-04154-s001.zip › ijms-1142860-supplementary.pdf]

Table S1. Analysis of the variants for alteration of splicing regulatory motifs. Negative ESRseq and Hexplorer scores indicates the decrease of exonic splicing enhancers` (ESE) or increase of exonic splicing silencers` (ESS) motifs. The higher EX-SKIP value is, the more ESS prevail over ESE in mutated sequence. ESE motifs favor exon recognition, while ESS motifs inhibit it.

Variant (*PCCA*)	HExoSplice (ESRseq score variant vs wild-type)	EX-SKIP (ESS/ESE of mutated sequence to ESS/ESE of WT sequence)	Hexplorer Score delta (mt-wt)
c.431G>T (p.Gly144Val)	-2.5128	1,57	-95.62
c.611_613del (p.Glu230del)	N/A	1,43	-105.6
c.742G>A (p.Glu248Lys)	-3.7007	1,24	-97.31
c.893A>G (p.Lys298Arg)	-1.4216	1,21	-124
c.1103A>G (p.Asp368Gly)	-1.9296	1,28	-110
c.1292T>G (p.Val431Gly)	-1.3075	1,26	-80.58
c.2056G>T (p.Glu686*)	-3.8884	1,32	-134
c.1367G>T (p.Gly456Val)	+0.3858	1,65	-46.07
